# Polymorphism of SLC6A2 gene does not influence outcome of myocardial ^123^I-*m*IBG scintigraphy in patients with chronic heart failure

**DOI:** 10.1007/s12350-016-0722-x

**Published:** 2016-11-14

**Authors:** Derk O. Verschure, F. Baas, Berthe L. F. van Eck-Smit, G. Aernout Somsen, Hein J. Verberne

**Affiliations:** 1 Department of Nuclear Medicine, Academic Medical Center, University of Amsterdam, P.O. Box 22700, 1100 DE Amsterdam, The Netherlands; 2 Department of Cardiology, Zaans Medical Center, Zaandam, The Netherlands; 3 Department of Genome Analysis, Academic Medical Center, University of Amsterdam, Amsterdam, The Netherlands; 4 Cardiology Centers of the Netherlands, Amsterdam, The Netherlands

**Keywords:** Cardiac sympathetic activity, planar ^123^I-*m*IBG myocardial scintigraphy, polymorphism, norepinephrine transporter, SLC6A2 gene

## Abstract

**Aim:**

The NET, encoded by SLC6A2, is responsible for presynaptic NE-reuptake. ^123^I-*m*IBG is clinically used to evaluate cardiac sympathetic function. However, it is unknown if polymorphism of SLC6A2 influences cardiac sympathetic activity as assessed with ^123^I-*m*IBG. Therefore we studied the influence of SLC6A2 SNPs on myocardial ^123^I-*m*IBG parameters in CHF.

**Materials and Methods:**

Forty-nine adults with stable CHF (age 66.5 ± 8.1 years, LVEF 22.3 ± 6.4) were enrolled. Fifteen minutes (early) and 4 hours (late) after administration of ^123^I-*m*IBG planar images were acquired. The H/M ratio was calculated from the manually drawn ROI over the left ventricle and a fixed mediastinal ROI. Fourteen exons of the SLC6A2 gene were analyzed from whole blood samples.

**Results:**

We found 6 different SLC6A2 SNPs, although none were functional. LVEF was the only independent predictor for early (adjusted *R* ^2^ = 0.063, *p* = 0.045) and late H/M ratio (adjusted *R* ^2^ = 0.116, *p* = 0.010). NT-proBNP was the only independent predictor for ^123^I-*m*IBG WO (adjusted *R* ^2^ = 0.074, *p* = 0.032). SLC6A2 SNPs were not associated with any myocardial ^123^I-*m*IBG-derived parameter.

**Conclusion:**

In this specific CHF population polymorphism of SLC6A2 gene was not associated with any ^123^I-*m*IBG derived parameters.

## Introduction

**Figure 1:**
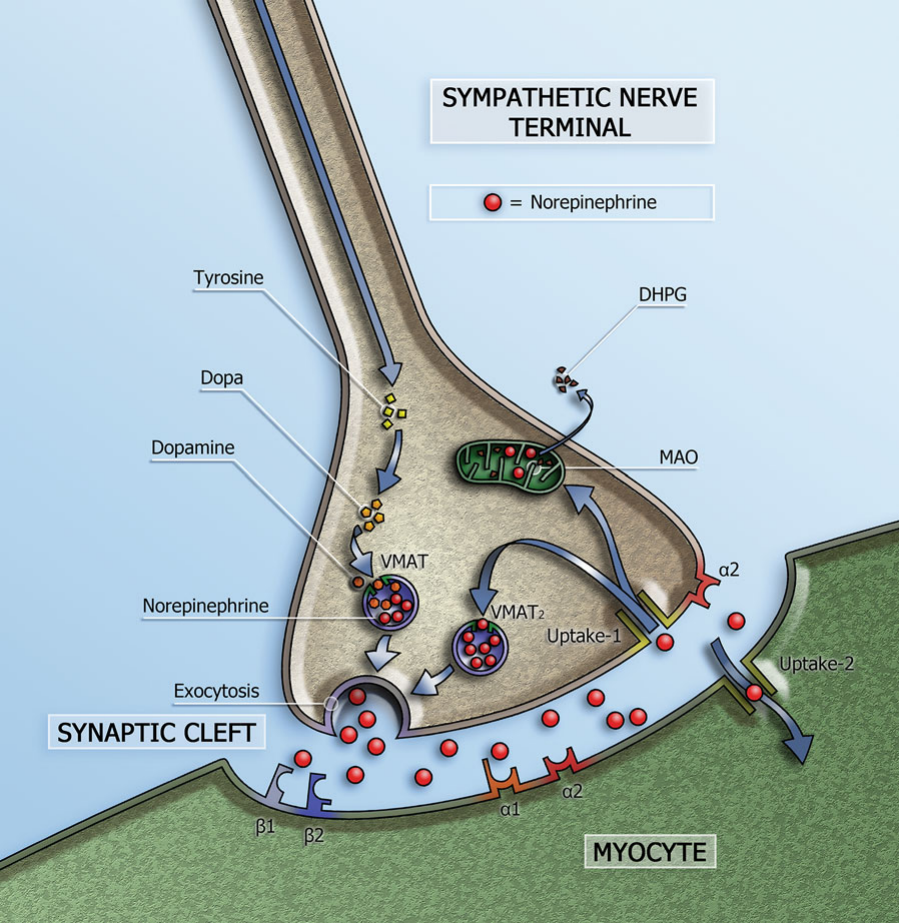
Schematic representation of the sympathetic synapse. Norepinephrine is synthesized within neurons by an enzymatic cascade. Dihydroxyphe-nylalanine (DOPA) is generated from tyrosine and subsequently converted to dopamine by DOPA decarboxylase. Dopamine is transported into storage vesicles by the energy-requiring vesicular monoamine transporter (VMAT). Norepinephrine is synthesized by dopamine β-hydroxylase within these vesicles. Neuronal stimulation leads to norepinephrine release through fusion of vesicles with the neuronal membrane (exocytosis). Apart from neuronal stimulation, release is also regulated by a number of presynaptic receptor systems, including α2–adrenergic receptors, which provide negative feedback for exocytosis. Most norepinephrine undergoes reuptake into nerve terminals by the presynaptic norepinephrine transporter (uptake-1 mechanism) and is re-stored in vesicles (following uptake by vesicular amine transporter 2 (VMAT2)) or is metabolized in cytosol dihydroxyphenylglycol (DHPG) by monoamine oxidase (MAO)

NE is the neurotransmitter of the cardiac sympathetic system and is stored in vesicles in the presynaptic nerve terminals (Figure 1). On the basis of tissue NE content, the heart is characterized by dense sympathetic innervation with a gradient from atria to base of the heart and from base to apex of the ventricles.^1^ Only a small amount of the released NE in the synaptic cleft is available to stimulate the post-synaptic β-AR on the myocytes. Most of the NE undergoes reuptake into the nerve terminals via uptake-1 mechanism. This transport system, i.e. NET, is sodium- and chloride-dependent and responsible for approximately 70% to 90% of the NE re-uptake from the sympathetic cleft.^2^ Genetic or acquired defects of the NET could affect the NE homeostasis and cause alterations in synaptic NE levels with consequent alterations in β-AR stimulation. The NET is a member of SLC6A2 and is encoded by the SLC6A2 gene^3^ located on human chromosome 16q12.2.^4^ This gene is encoded by 16 exons which span 45 kb from the start to the stop codon.^5^ SNPs of the SLC6A2 gene which result in amino acid substitutions have been reported. Many of these variations were derived from specific psychiatric and cardiovascular phenotypes and only a limited number have been examined for alterations in function.^6^–^9^ In a familial form of idiopathic POTS a SNP of the SLC6A2 gene in exon 9 that resulted in loss of function of the NET was associated with increased NE plasma levels and increased heart rate.^10^,^11^

The cardiac sympathetic system is one of the neurohormonal compensation mechanisms that plays an important role in the pathogenesis of chronic heart failure (CHF). Patients with CHF have increased cardiac sympathetic activity with increased exocytosis of NE from the presynaptic vesicles, as well as increased plasma and urinary levels of NE concomitant with the severity of left ventricular dysfunction.^12^–^14^ In addition, the NE re uptake via the NET is decreased resulting in elevated synaptic levels of NE. Initially, β-adrenergic receptor stimulation by increased NE levels helps to compensate for impaired myocardial function, but long-term NE excess has detrimental effects on myocardial structure and gives rise to a down regulation of post-synaptic β-adrenergic receptors.^15^ This down regulation leads to left ventricle remodeling and poor prognosis.

^123^I-*m*IBG, a NE analog, shares the same presynaptic uptake, storage and release mechanisms as NE. Radiolabeling of *m*IBG with ^123^I allows imaging with gamma cameras.^16^ Myocardial ^123^I-*m*IBG scintigraphy is a reliable non-invasive imaging technique to assess cardiac sympathetic activity and has been shown to be of clinical value, especially for the assessment of prognosis, in many cardiac diseases.^17^–^20^ However, there are several factors that influence the cardiac ^123^I-*m*IBG-derived parameters (e.g. choice of collimator and acquisition duration). It is conceivable that polymorphisms of the SLC6A2 gene might also influence these cardiac ^123^I-*m*IBG derived parameters. Therefore, the aim of this study was to investigate the relation between polymorphisms of the SLC6A2 gene and pre synaptic NE uptake in CHF patients as assessed with myocardial ^123^I-*m*IBG scintigraphy.

## Material and Methods

### Subjects

Subjects with stable CHF eligible for implantable cardioverter device (ICD) implantation for primary prevention of sudden cardiac death, who were referred for ^123^I-*m*IBG scintigraphy to the department of nuclear medicine of the Academic Medical Center, in the period December 2010 - September 2015, were asked to participate. The principal study inclusion criteria were both ischemic and non-ischemic heart failure patients with New York Heart Association (NYHA) functional class II or III and LVEF <35% as assessed with echocardiography. All subjects were treated with optimal medical therapy according to the European heart failure guidelines, including beta blockers and angiotensin-converting-enzyme inhibitors (ACE-I) or angiotensin receptor blockers (ARB) and when necessary loop diuretics.^21^ Exclusion for participation was pregnancy or intolerance for iodine. The study was approved by the local institutional review board and conducted according to the principles of the International Conference on Harmonization–Good Clinical Practice.

### Genotyping

The deoxyribonucleic acid (DNA) of the subjects was extracted from whole blood samples using standard protocols. In total 14 exons of the SLC6A2 gene were analyzed by Sanger sequencing using BigDye terminator chemistry on a 3730XL capillary sequencer. Sequence traces were analyzed in Codoncode Aligner software with the reference sequence NM: 001172504.1. Analysis was performed by an experienced observer blinded to patient data. The sequence variants were analyzed for predicted effect on splicing using the Alamut software suite (Interactive Biosystems, France).

### ^123^I-*m*IBG Scintigraphy Acquisition and Analysis

To block uptake of free ^123^I by the thyroid gland, subjects were pre-treated with 250 mg oral potassium iodide 30 min before intravenous (IV) injection of 185 MBq^123^I-*m*IBG. Fifteen minutes (early acquisition) and 4 hours (late acquisition) after administration of ^123^I-*m*IBG, 10-min planar images were acquired with the subjects in supine position using a medium energy collimator.

**Figure 2:**
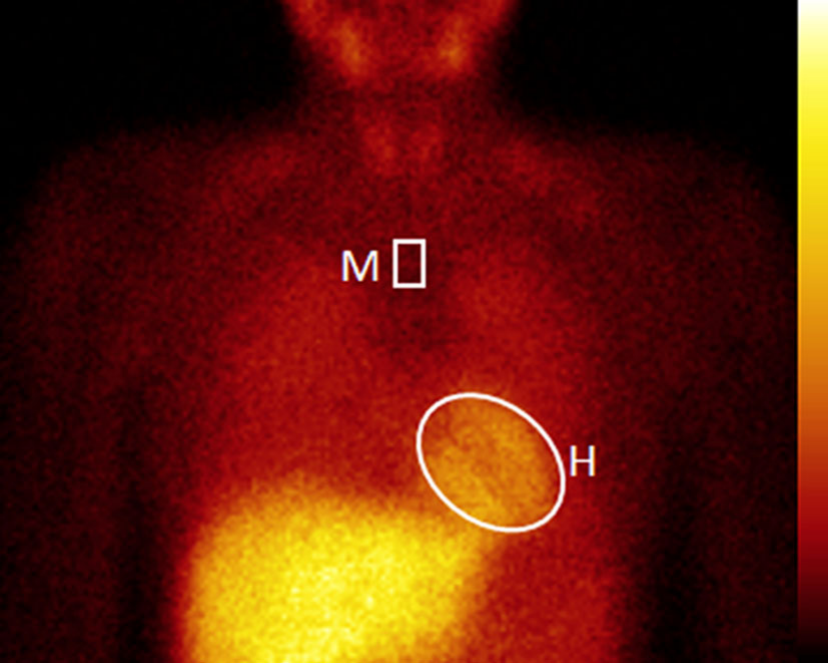
Example of post processing planar ^123^I-*m*IBG images. The positioning of the mediastinum ROI (M) is determined in relation to the lung apex, the lower boundary of the upper mediastinum, and the midline between the lungs. The manually drawn cardiac ROI (H) is placed over the myocardium including the left ventricular cavity

All planar ^123^I-*m*IBG images were analyzed by one experienced observer (D.O.V.) blinded to patient data. H/M ratios were calculated from the ^123^I-*m*IBG images using a ROI over the heart and the upper part of the mediastinum. The cardiac ROI was manually drawn over the myocardium including the left ventricular cavity. A fixed rectangular mediastinal ROI was placed on the upper part of the mediastinum.^22^ The location of the mediastinal ROI was determined in relation to the lung apex, the lower boundary of the upper mediastinum, and the midline between the lungs (Figure 2). The H/M ratio was calculated by dividing the mean count density in the cardiac ROI by the mean count density in the mediastinal ROI.^22^ The ^123^I-*m*IBG WO was calculated using the early and late H/M ratios with the following formula:

$$ {\text{WO }} = \left\{ {\frac{{({\text{early }}\,H/M\,{\text{ ratio}}) - ({\text{late }}\,H/M\,{\text{ ratio}})}}{{{\text{early }}H/M{\text{ ratio}}}}} \right\}\, \times 100 $$

The H/M ratio reflects presynaptic uptake of ^123^I-*m*IBG. The early H/M ratio reflects predominantly the integrity of sympathetic nerve terminals (i.e., number of functioning nerve terminals and intact uptake-1 mechanism). The late *H*/*M* ratio offers predominantly information about neuronal function resulting from uptake, storage and release. The ^123^I-*m*IBG WO reflects predominantly neuronal integrity of sympathetic tone/adrenergic drive.

### Statistical Analysis

All continuous variables are expressed as a mean ± standard deviation. After demonstrating a normal distribution of variables, between-group comparisons were performed by using independent-sample *t* tests. Differences between groups for continuous data were compared using analysis of variance (ANOVA). Multivariate regression analysis was performed to determine independent predictors of ^123^I-*m*IBG outcomes. Haplotype, genotype (the combination of 2 haplotypes), LVEF, NT-proBNP and functional class NYHA were used as explanatory variables. The overall goodness of fit for each model was expressed as the adjusted *R* ^2^. A *p* value <.05 was considered to indicate a statistically significant difference. Statistical analyses were performed with SPSS, release 22.0 for Windows (SPSS Inc., Chicago, IL, USA 2003).

## Results

### Subjects

Table 1 shows the characteristics of the study population. A total of 49 CHF subjects (80% men) were enrolled with a mean age of 66 ± 8 years and a mean LVEF of 22.3% ± 6.4%. The mean early H/M ratio was 2.11 ± 0.39, late H/M ratio was 1.81 ± 0.39, and ^123^I-*m*IBG WO was 13.8% ± 11.2%.

**Table 1:** Baseline characteristics CHF patients

	Total (*n* = 49)
Age (years)	66 ± 8
Sex, male (%)	39 (80)
Body mass index (kg/m^2^)	27.5 ± 4.4
LVEF (%)	27.5 ± 4.4
Heart rate (beats/min)	76 ± 15
Systolic BP (mmHg)	127 ± 18
Diastolic BP (mmHg)	77 ± 11
NYHA functional class	
II (%)	36 (73)
III (%)	13 (27)
Etiology heart failure	
Ischemic (%)	28 (57)
Non-ischemic (%)	21 (43)
Medical history	
Hypertension (%)	23 (47)
Diabetes (%)	13 (27)
Laboratory results	
NT-pro BNP (ng/L)	2109 ± 3169
^123^I-*m*IBG WO scintigraphy	
Early H/M ratio	2.11 ± 0.39
Late H/M ratio	1.81 ± 0.39
^123^I-*m*IBG WO	13.8 ± 11.3

### Genotyping

**Figure 3:**
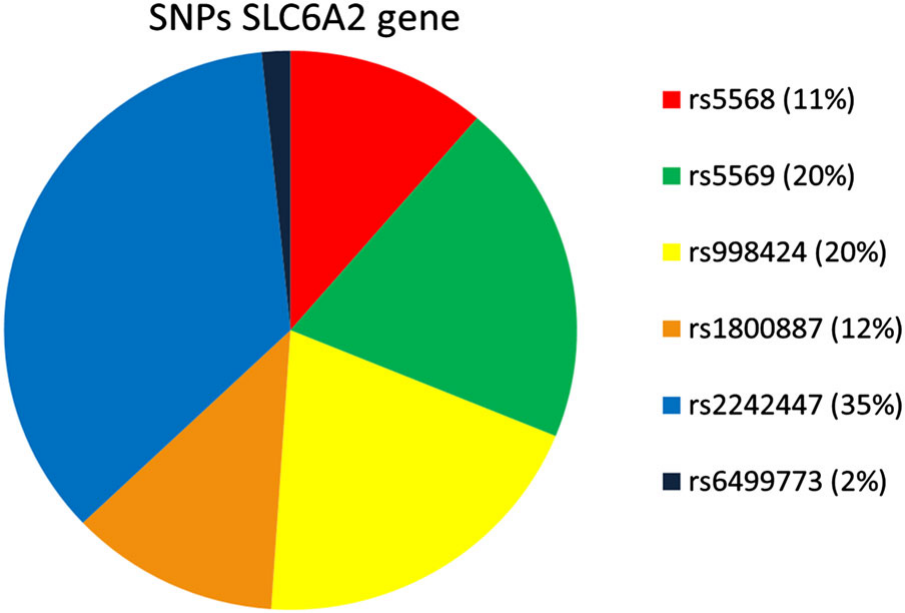
Relative contribution (%) of the six different single-nucleotide polymorphisms (SNPs) of the SLC6A2 gene in the study population (*n* = 49)

**Figure 4:**
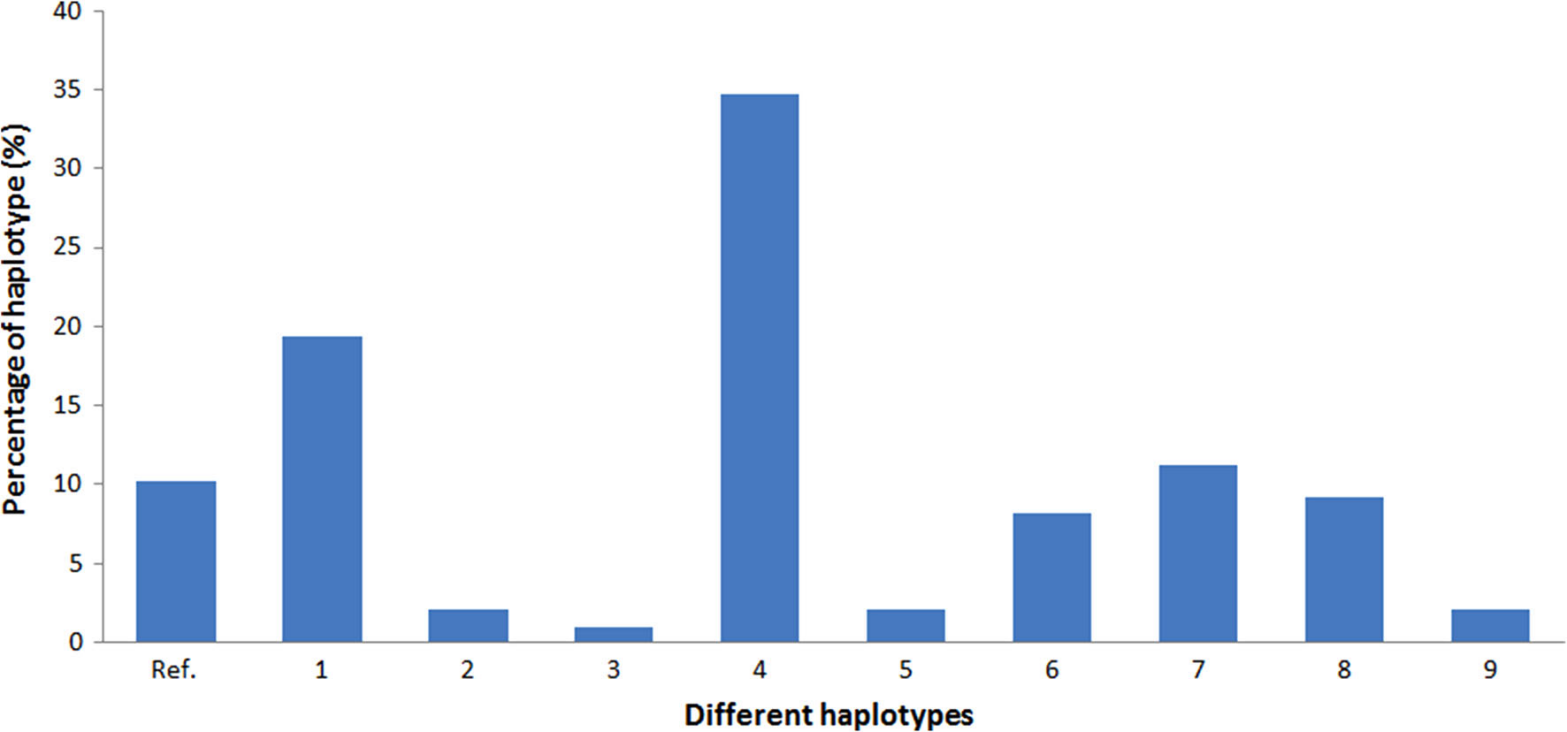
Relative contribution of haplotypes in 49 CHF patients including two times 49 alleles (*n* = 98). Ref. = reference allel without any SNPs, 1 = rs5568; rs2242447, 2 = rs5568; rs5569; rs998424; rs2242447, 3 = rs5569; rs998424; rs1800887; rs6499773, 4 = rs5569; rs998424; rs2242447, 5 = rs5569; rs1800887; rs2242447, 6 = rs1800887, 7 = rs1800887; rs6499773, 8 = rs2242447, 9 = rs6499773

Analysis of the SLC6A2 gene showed 6 different SNPs in 47 subjects (in 2 subjects no SNPs were found): c.1148-13A > C (rs5568), c.1287G > A p.Thr429Thr (rs5569), c.1389 + 9G > A (rs998424), c.1590 + 23T > C (rs1800887), c.1830 + 66C > T (rs2242447), c.1831-122T > A (rs6499773) (Figure 3). Only SNP rs5569 was located in an exon and was synonymous. All other SNPs were located in a non-coding area. None of the SNPs were functional (i.e. causing a change in amino acid or affecting splicing). In this study population ten different haplotypes could be constructed from the 6 different SNPs (Figure 4) resulting in 22 different genotypes. The alleles of two SNPs, rs5568 and rs2242447, showed linkage disequilibrium. Another fixed inherited combination of the SNPs is rs5569, rs998424 and rs2242447 showing high linkage disequilibrium.

### Multivariate Regression Analysis

Multivariate regression analysis using haplotype, genotype, LVEF, NT-proBNP, and functional NYHA class did not show any relation of haplotype or genotype with early and late H/M ratios nor ^123^I-*m*IBG WO. LVEF was the only independent predictor of early H/M ratio (adjusted *R* ^2^ = 0.063, *p* = 0.045) and late H/M ratio (adjusted *R* ^2^ = 0.116, *p* = 0.010). (Table 2) In addition, NT-proBNP was the only independent predictor for ^123^I-*m*IBG WO (adjusted *R* ^2^ = 0.074, *p* = 0.032).

**Table 2:** Multivariate analysis of possible independent predictors of early H/M ratio, late H/M ratio and ^123^I-*m*IBG WO (*n* = 49)

^123^I-*m*IBG parameter	Independent predictor	Adjusted *R* ^2^	*p* value
Early H/M ratio	LVEF	0.063	0.045
Late H/M ratio	LVEF	0.116	0.010
^123^I-*m*IBG WO	NT-pro BNP	0.074	0.032

## Discussion

To the best of our knowledge this is the first time the relationship between SLC6A2 polymorphism and cardiac sympathetic activity has been studied. Although 6 SNPs of the SLC6A2 gene were found in this study, there was no relationship between these SNPs and cardiac sympathetic activity as assessed with ^123^I-*m*IBG.

The ME-collimator-derived mean early and late H/M ratios in this CHF population were lower compared to ME-collimator-derived mean early and late H/M ratios in healthy subjects. Recently, corrected mean values for ME-collimator-derived early and late HM ratios in healthy subjects have been reported (3.1 [2.2-4.0] and 3.3 [2.2-4.4], respectively).^23^ Compared to other CHF populations, ^123^I-*m*IBG WO was relatively low.^20^,^24^ In part, this may be related to differences in WO calculation. However, it may also be a reflection of the stable condition of our patients.

Functional missense mutation in the SLC6A2 gene (Ala457Pro) resulting in only 2% of the NET activity with consequent increase in NE plasma levels has been reported in a familial form of POTS.^10^ In addition, inhibition of NE uptake with atomoxetine worsens the symptom burden in subjects with POTS suggesting the important role of NE uptake in this syndrome.^25^ In essential hypertension myocardial NE uptake is impaired.^26^–^28^ Although hypertension is multifactorial it is conceivable that functional SLC6A2 SNPs affect blood pressure.^9^,^29^ SNPs of SLC6A2 have been identified and, only rs168924 was associated with the incidence of essential hypertension.^30^ The discovery of the linkage with SLC6A2 gene mutations in POTS and hypertension resulting in decreased NE uptake activity suggests that a faulty NET may lead to an impaired cardiac ^123^I-*m*IBG uptake. Interestingly, there are differences between different organs in NE spillover. In general, the myocardial NE re uptake is very efficient and only 2% to 3% of the systemic NE spillover (i.e. plasma) can be attributed to myocardial origin.^31^ As NE re uptake mainly depends on NET, these data suggest that the myocardial SLC6A2 (i.e. NET) expression/activity level is higher compared to other tissues.

We assumed that polymorphism of the SLC6A2 gene could influence the NE uptake and consequently explain variation in the ^123^I-*m*IBG-derived parameters. In this CHF population there were 6 SNPs. Although most of these SNPs occur frequently (Table 3), none of these SNPs caused a change in amino acid or affect splicing. Therefore it was not surprising that variation in early H/M ratio, late H/M ratio or ^123^I-*m*IBG WO could not be explained by the different haplotypes.

**Table 3:** Frequency (%) worldwide and in Europe of the 6 SNPs founded in our study population.^33^

Haplotype	Worldwide	Europe
rs5568	74.8	64.4
rs5569	76.7	64.0
rs998424	77.1	64.0
rs1800887	71.0	78.4
rs2242447	52.3	31.2
rs6499773	80.8	85.1

LVEF and NT-proBNP were moderately, but significantly related to ^123^I-*m*IBG-derived parameters. It has been shown that BNP modulates autonomic nervous function by inhibiting cardiac sympathetic activity in CHF.^32^ As in CHF, prolonged increased cardiac sympathetic activity has a detrimental effect on the contractility of the myocardium and thereby negatively influences the LVEF.

Our study has some limitations. The sample size of the study is relatively small and may have resulted in a limited number of different haplotypes and statistical powers. In addition, the SNPs identified in our study were not functional (i.e., no change in amino acid). Therefore, the effect of functional SNPs of the SLC6A2 gene to cardiac sympathetic activity assessed by ^123^I-*m*IBG scintigraphy remains unanswered.

The results of this study suggest that SNPs of SLC6A2 at non-slice sites do not affect the ^123^I-*m*IBG uptake. Consequently, polymorphism of SLC6A2 is not a confounder of the myocardial ^123^I-*m*IBG scintigraphy-derived parameters in this population. However, extrapolation of these findings to the overall CHF population should be done with care.

In conclusion, the results of this study showed that in this specific CHF population the variation in ^123^I-*m*IBG scintigraphy-derived parameters cannot be explained by polymorphism of the SLC6A2 gene.

## New Knowledge Gained

The current data suggest that functional polymorphism of the SLC6A2 gene seems less common in our CHF population compared to patients with hypertension or POTS. In addition, ^123^I-*m*IBG-derived parameters are more related to common prognostic parameters, such as LVEF and NT-proBNP, than polymorphism of the SLC6A2 gene.

## Abbreviations

β-AR: β-adrenergic receptors
H/M: Heart-to-mediastinum
NE: Norepinephrine
NET: Norepinephrine transporter
NT-proBNP: N-terminal pro B-type Natriuretic Peptide
SLC6A2: Solute carrier family 6
SNP: Single-nucleotide polymorphism
POTS: Postural orthostatic tachycardia syndrome
ROI: Region of interest
WO: Washout
